# Temperature Mapping During MR‐Guided Cryoablation Using a FLORET UTE Sequence

**DOI:** 10.1002/mrm.70152

**Published:** 2025-10-24

**Authors:** Moritz Gutt, Othmar Belker, Julienne Scheller, Simon Schröer, Frank Wacker, Bennet Hensen, Marcel Gutberlet

**Affiliations:** ^1^ Institute of Diagnostic and Interventional Radiology Hannover Medical School Hannover Germany; ^2^ Research Campus STIMULATE Magdeburg Germany

**Keywords:** cryoablation, FLORET, MR thermometry, ultrashort‐echo imaging

## Abstract

**Purpose:**

To implement a three‐dimensional FLORET‐UTE sequence for rapid MR thermometry during cryoablation and to validate voxel‐wise temperature maps against fiber‐optic probes in ex vivo bovine liver.

**Methods:**

A FLORET‐UTE trajectory (TE 50 μs, 1.6 mm isotropic, 18 s/volume) was executed on a 3 T scanner while dry‐ice cryoablation was applied to liver phantoms. Four fiber‐optic sensors provided reference temperatures. Magnitude signal was normalized to baseline and fitted to a mono‐exponential temperature model in the first experiment; the unchanged model was applied prospectively to the second dataset. Images were reconstructed offline with $\ell_{2}$‐regularized non‐Cartesian SENSE. Accuracy below 0°C was assessed with root‐mean‐square error (RMSE) and Bland–Altman analysis.

**Results:**

Calibration yielded a strong signal–temperature relationship ($R^{2}=0.95$). Prospective validation produced RMSEs of 0.93°C–1.73°C (overall 1.33°C). Bland–Altman bias was −0.08°C with limits of agreement −2.08°C to 2.23°C. Serial temperature maps captured three‐dimensional ice‐ball dynamics at 18‐s temporal resolution—substantially faster than previously reported radial‐UTE and STIR‐UTE approaches.

**Conclusion:**

FLORET‐UTE enables quantitative 3‐D MR thermometry of frozen tissue with < 20 s temporal resolution and < 2°C accuracy, overcoming the speed limitations of earlier UTE methods. The technique is a promising candidate for real‐time MR‐guided cryoablation and warrants in vivo evaluation.

## 1 Introduction

Cryoablation has been established as a minimally invasive treatment technique in which tissue necrosis is induced through the application of sub‐zero temperatures. It has been utilized in the treatment of tumors in organs such as the kidney, liver, lung, and prostate [1, 2, 3, 4]. In these applications, magnetic resonance imaging (MRI) has frequently been employed for procedural guidance, owing to its excellent soft tissue contrast and its ability to visualize the iceball formed during ablation [5, 6, 7].

While most MR sequences visualize the iceball margin, this boundary does not correspond to the lethal isotherm (≤ −20°C) required for complete tumor necrosis, since the true ablation zone is substantially smaller than the iceball, potentially leading to overestimation of the treated area [8, 9, 10, 11]. Therefore, direct intraprocedural thermometry to delineate the lethally ablated zone is desirable.

In thermal ablation procedures involving heat, MR thermometry, particularly proton resonance frequency (PRF) shift‐based methods, has been widely implemented for real‐time temperature mapping. However, these techniques are ineffective in frozen tissue due to substantial signal loss arising from extremely short $T_{2}^{*}$ relaxation times [12].

To address this limitation, ultrashort echo time (UTE) sequences have been proposed. These sequences enable the acquisition of signal from tissues with short $T_{2}^{*}$ values and have thus been explored for temperature monitoring during cryoablation. Overduin et al. [13] demonstrated that quantitative temperature mapping could be achieved using radial UTE imaging by exploiting the temperature dependence of signal magnitude in frozen tissue. Alternatively, Tokuda et al. [14] proposed a STIR‐UTE approach to visualize lethal isotherms based on $T_{1}$‐weighted contrast. While their method enabled qualitative delineation of regions within a specific temperature range, quantitative temperature estimation was not performed. Both methods required scan times exceeding 1 min per volume, limiting their feasibility for real‐time monitoring in a clinical context.

As an alternative approach, Krishnamoorthy et al. [15] proposed a slab‐selective stack‐of‐spirals sequence which could achieve scan times of 20–30 s. However, their approach comes with a smaller field of view and lower resolution along the z‐axis (140 mm FOV and 6 mm slice thickness), incoherent echo times along the $k_{z}$‐axis and a higher echo time in general (minimum of 160 μs at $k_{z}=0$).

The FLORET [16] (Fermat Looped, Orthogonally Encoded Trajectories) UTE sequence has been developed to provide efficient 3D k‐space sampling with center‐out trajectories and ultrashort echo times. This trajectory design has been shown to offer improved sampling efficiency over radial acquisitions used in prior studies, thereby enabling higher temporal resolution. These characteristics suggest that FLORET may be more suitable for integration into clinical workflows requiring near real‐time thermometry.

The aims of this study were to (i) implement a FLORET‐UTE acquisition and reconstruction pipeline for frozen tissue, (ii) establish a voxel‐wise signal–temperature calibration in ex vivo bovine liver, and (iii) validate the resulting temperature maps against fiber‐optic probes.

## 2 Methods

### 2.1 FLORET Sequence Design

FLORET is a 3D spiral k‐space trajectory designed to provide efficient center‐out coverage with ultrashort echo times. The sequence is constructed by projecting a 2D Fermat spiral onto conical shells oriented along multiple axes, producing sets of interleaves referred to as “hubs” [16]. Within each hub, the Fermat spiral is rotated by the golden angle to ensure uniform k‐space sampling.

In this study, three hubs were employed (maximum cone angle 36°), and each hub consisted of 2924 interleaves. To improve gradient fidelity and minimize system‐induced artifacts, the base spiral was designed using a frequency‐constrained waveform, as described by Pipe and Borup [17]. This method suppresses high‐frequency components in the gradient waveform while preserving the intended trajectory, enhancing trajectory accuracy especially at the central part of k‐space.

### 2.2 Experimental Setup

Experiments were conducted using an ex vivo phantom composed of bovine liver tissue. Cryoablation was performed by delivering dry ice pellets through a funnel system connected to a plastic tube, directing the pellets into a cavity within the phantom.

Four fiber‐optic temperature sensors (FOTEMPOEM‐PLUS, 200 μm fiber, 300 μm × 300 μm sensitive tip area, Weidmann Technologies Deutschland GmbH) were inserted into the tissue and fixed in position. A high‐resolution scan was acquired before freezing to localize the sensors, which were then registered manually to the 3D reconstruction grid. Each experiment began with a 3‐min baseline acquisition, followed by continuous cryogen delivery over a 30‐min scan duration. Approximately 100 volumetric images were acquired during the whole scan.

**FIGURE 1:**
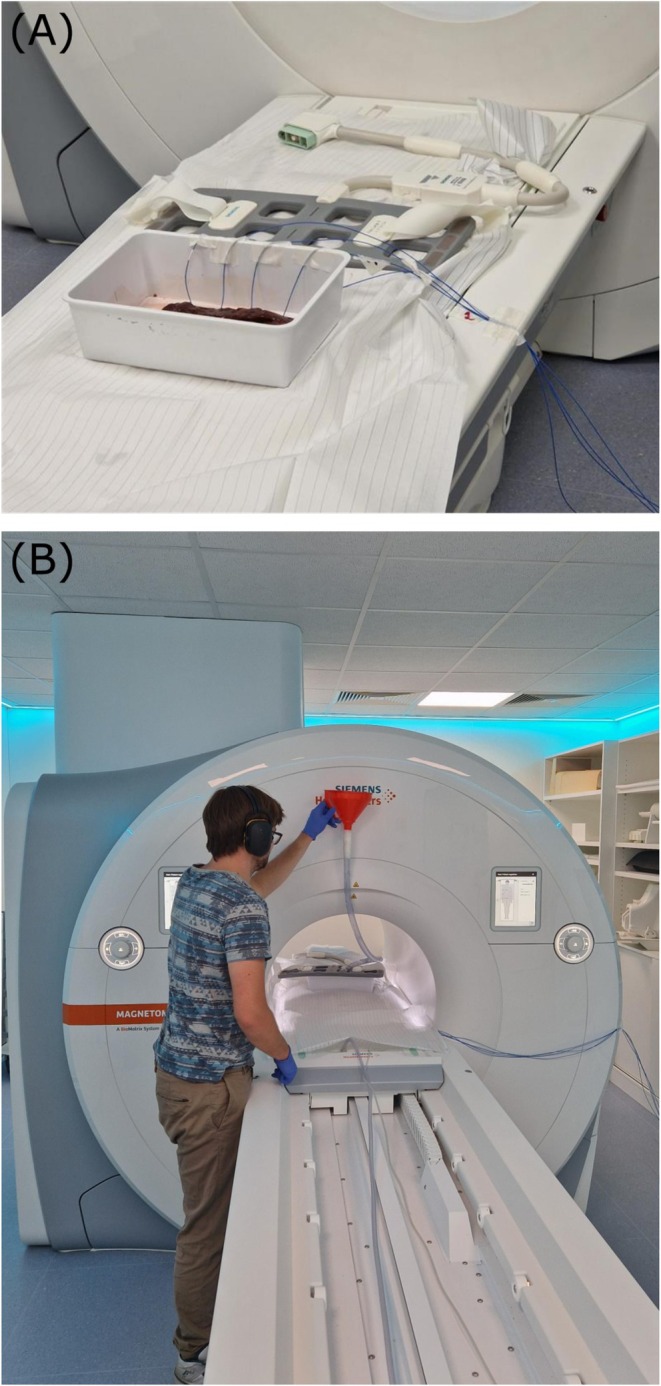
Experimental setup: (A) liver phantom with the optical temperature sensors; (B) application of the dry ice through the funnel and plastic tube.

Images of the experimental setup are shown in Figure 1.

### 2.3 MR Imaging Protocol

All imaging was performed on a 3 T Siemens MAGNETOM Vida clinical scanner (Siemens Healthineers, Erlangen, Germany). A 20 μs nonselective rectangular RF excitation pulse was used, followed by a center‐out spiral readout based on the FLORET trajectory. Volumetric imaging was performed continuously throughout the freezing.

Imaging parameters were as follows:

- Echo time (TE): 50 μs.
- Repetition time (TR): 2 ms.
- Flip angle: 10°.
- Field of view (FOV): 320 mm (isotropic).
- Spatial resolution: 1.6 mm (isotropic).
- Temporal resolution per volume: 18 s.
- Readout duration: 1 ms.

### 2.4 Image Reconstruction

Image reconstruction was performed offline using the Berkeley Advanced Reconstruction Toolbox (BART). Coil sensitivity maps were estimated using the ncalib [18] function. Volumetric images were reconstructed iteratively using the pics algorithm. For the algorithm to reach convergence in a reasonable amount of iterations, it was necessary to add $\ell_{2}$‐regularization (λ=0.01) and k‐space preconditioning. The preconditioning method followed the approach described by Ong et al. [19], which applies a diagonal preconditioner to accelerate convergence in non‐Cartesian iterative reconstructions.

### 2.5 Temperature Calibration and Mapping

Voxel‐wise signal intensities were first normalized to the baseline (pre‐freeze) signal for each voxel. Temperature calibration was performed by fitting a mono‐exponential model to the normalized signal intensity and the corresponding sensor‐derived temperature values, using only time points where the sensors measured subzero temperatures:

$$S\left(T\right)=A\cdot e^{B\cdot T},$$

where S(T) is the normalized MR signal intensity, T is the temperature in degrees Celsius, and A and B are calibration parameters obtained by least‐squares fitting. This normalization and mono‐exponential fit are identical to Overduin et al. [13]. Residuals from the fit were examined to verify model adequacy. The fitted parameters were then applied voxel‐wise to generate 3D temperature maps for all imaging time points.

To evaluate reproducibility, the calibration model obtained from the first experiment was applied to a second, independently acquired dataset from a different specimen of the same ex vivo bovine liver. Temperature maps generated using this fixed model were compared with fiber‐optic sensor measurements to assess accuracy.

As movement cannot be completely avoided in this experimental setup due to the weight of the dry ice and possible shrinking of the frozen liver tissue (see Figure S1), a one‐time voxel selection was performed per sensor. During calibration, only the direct neighbors of the assumed sensor location were evaluated. The voxel with the highest $R^{2}$ was chosen and kept fixed for all frames. Likewise, for validation, the best‐matching directly neighboring voxel was chosen.

### 2.6 Data Analysis

All quantitative analyses were performed in Python. Signal normalization, exponential model fitting, and temperature conversion were conducted voxel‐wise.

Accuracy was evaluated using the root‐mean‐square error (RMSE) between MR‐derived and sensor‐measured temperatures from the validation experiment. Bland–Altman analysis was also performed to assess agreement. Only time points with subzero sensor readings were included in the calibration and validation analyses.

## 3 Results

### 3.1 Temperature Calibration

**FIGURE 2:**
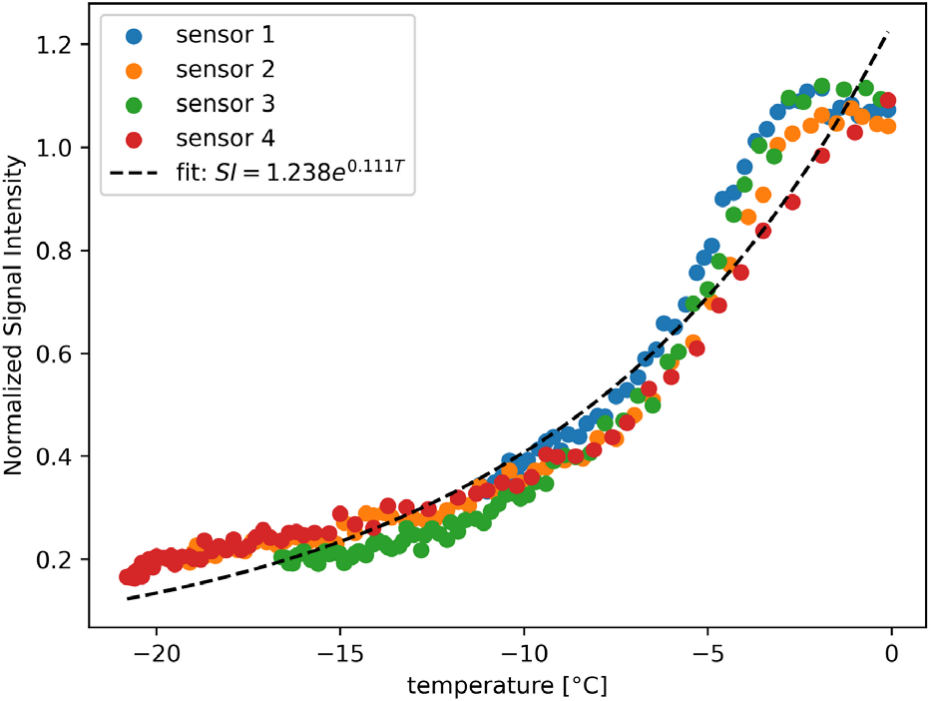
Calibration curve showing the exponential fit between normalized MR signal and temperature. Data points represent averaged signal intensity and corresponding sensor temperatures during the first (calibration) experiment. The fitted model achieved an $R^{2}$ of 0.95.

A mono‐exponential model was fitted to the normalized MR signal intensities and corresponding fiber‐optic sensor temperatures acquired during the first cryoablation experiment. It yielded *A* = 1.238 and *B* = 0.111°C^−1^. The resulting calibration model showed a strong exponential relationship between signal intensity and temperature, with a coefficient of determination of $R^{2}=0.95$. This model was subsequently applied voxel‐wise to the entire imaging volume to generate temperature maps over time. The fitted curve and calibration data are shown in Figure 2.

### 3.2 Validation Experiment

The application of the calibration model obtained from the first experiment to the validation experiment yielded Root‐mean‐square error (RMSE) values between MR‐derived and sensor‐measured temperatures for each of the four sensor locations (considering only subzero time points) of 1.73°C, 0.93°C, 1.16°C, and 1.18°C. The overall RMSE across all sensors and time points was 1.33°C.

**FIGURE 3:**
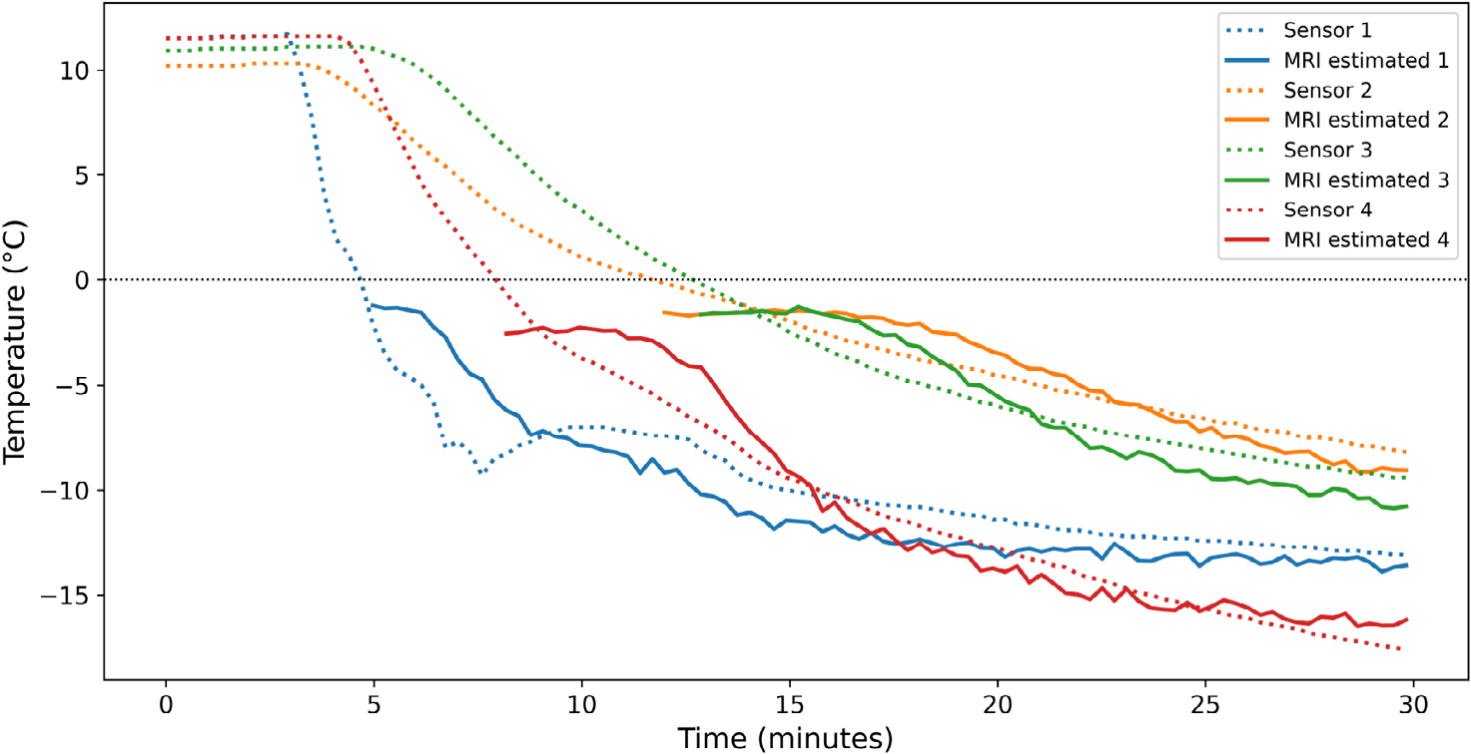
Comparison of MR‐estimated and sensor‐measured temperatures during the validation experiment. For the MR‐estimated temperatures, only subzero time points were included.

A plot comparing MR‐estimated and sensor‐measured temperatures is shown in Figure 3.

### 3.3 Temperature Map Visualization

Voxel‐wise temperature maps were reconstructed at each time point of the imaging series. These maps revealed the spatial expansion of the frozen region throughout the freezing.

**FIGURE 4:**
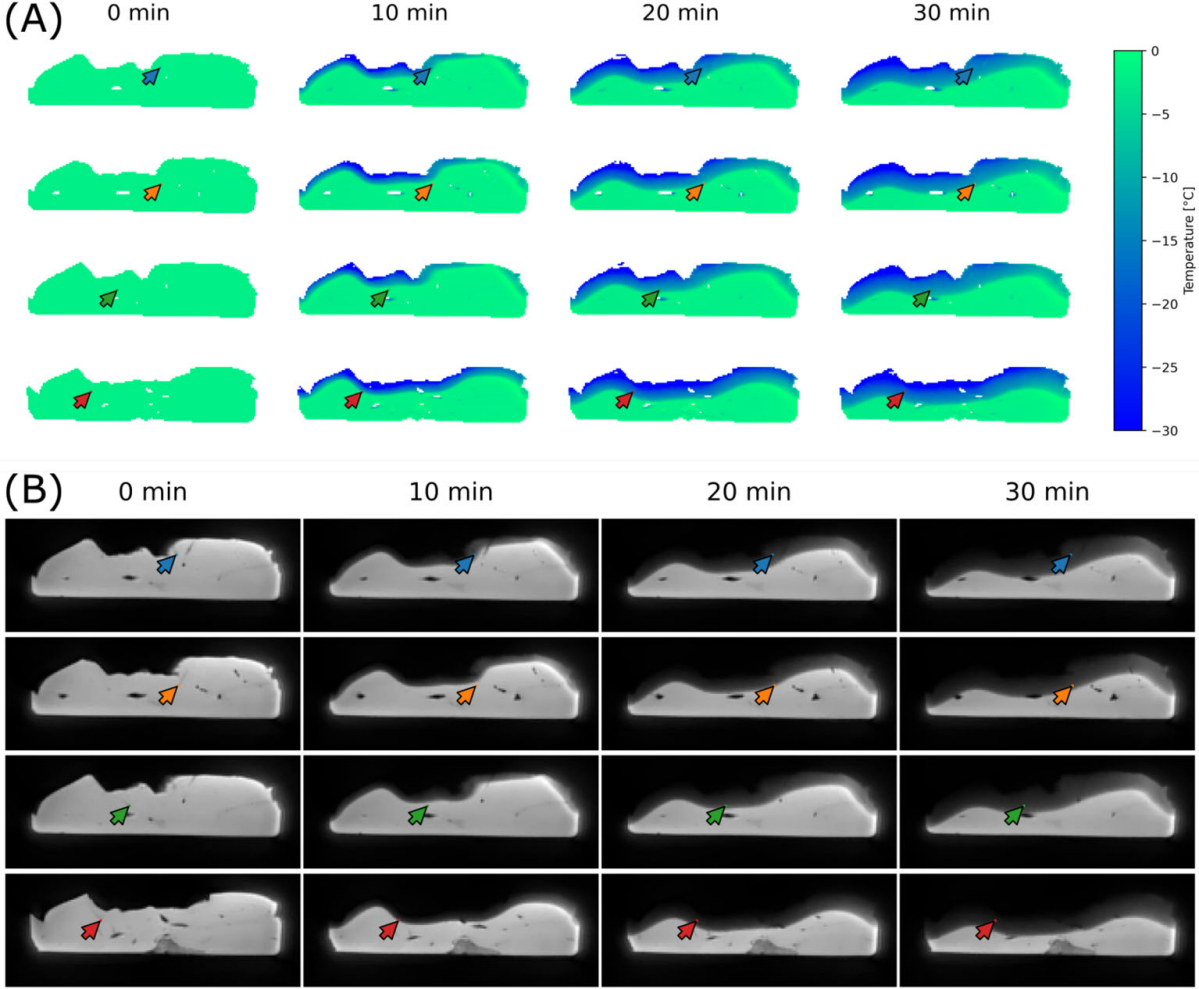
Axial slices at the location of the temperature sensors. (A) MRI‐estimated temperature maps and (B) corresponding magnitude images acquired at 0, 10, 20, and 30 min into the experiment. Sensor positions are indicated. Note that temperatures above 0°C cannot be estimated by the used method and appear to be close to 0°C.

Figure 4 shows axial slices at the location of the temperature sensors with calculated temperature maps and corresponding magnitude images at different time points in the experiment.

### 3.4 Agreement Analysis

**FIGURE 5:**
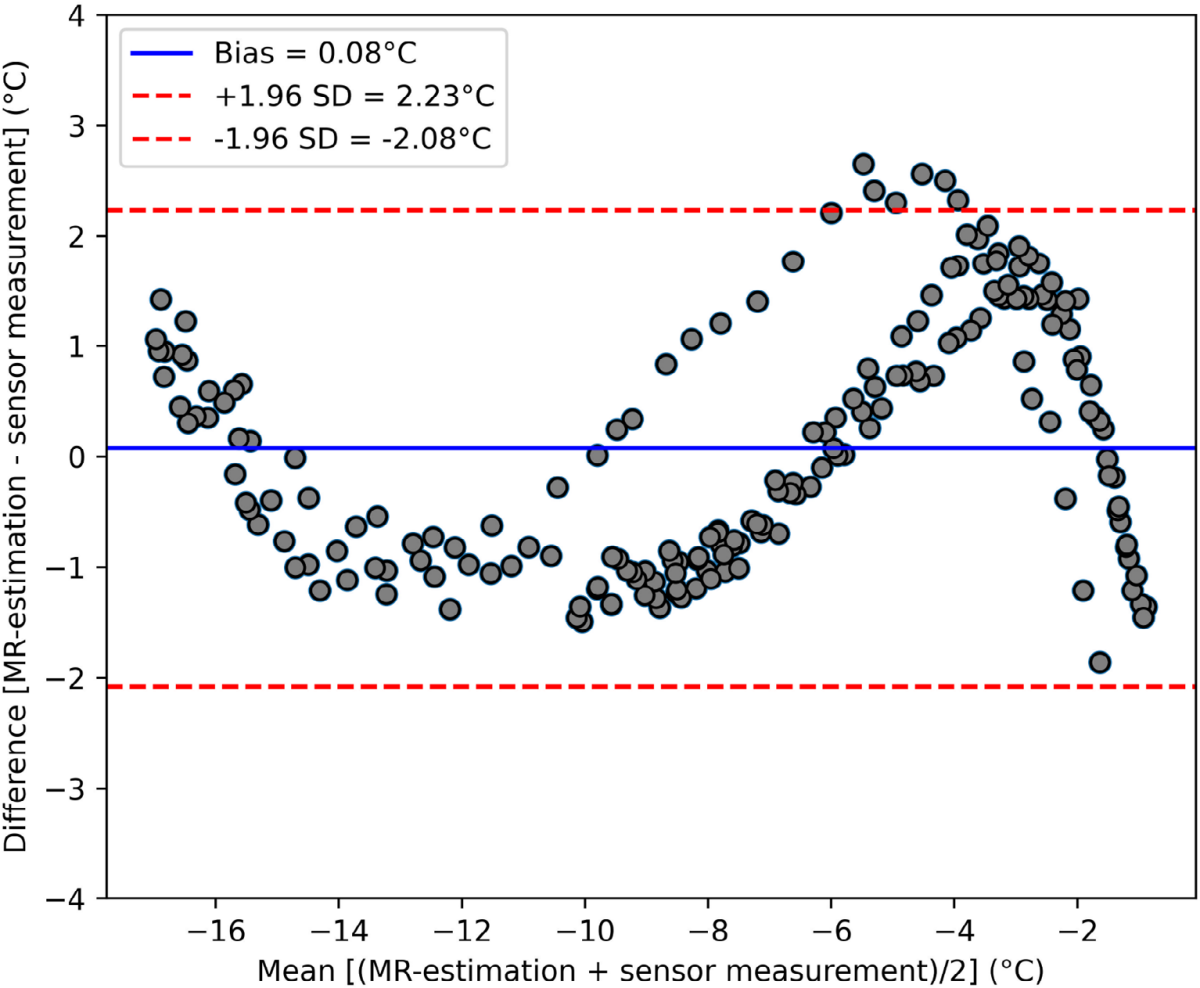
Bland–Altman plot showing agreement between MR‐estimated and sensor‐measured temperatures in the validation dataset. The mean bias was 0.08°C, with limits of agreement from −2.08°C to 2.23°C. Most differences fell within these limits.

A Bland–Altman analysis was performed on the validation dataset to assess agreement between MR‐estimated and sensor‐measured temperatures. The mean bias was −0.08°C, with limits of agreement ranging from −2.08°C to 2.23°C. The corresponding Bland–Altman plot is shown in Figure 5.

## 4 Discussion

This study demonstrated the feasibility of using a FLORET UTE sequence for temperature mapping during MR‐guided cryoablation. Quantitative temperature maps were generated from normalized signal intensities using a voxel‐wise exponential model, and the resulting temperature estimates showed good agreement with fiber‐optic sensor measurements in an ex vivo bovine liver model.

Compared to previous work by Overduin et al. [13] employing a radial UTE acquisition, the FLORET sequence enabled substantially higher temporal resolution (18 s per volume vs. > 1 min), while maintaining adequate signal‐to‐noise ratio for temperature calibration. The spiral‐based FLORET trajectory, combined with frequency‐constrained gradient design, allowed for efficient center‐out k‐space sampling with reduced gradient‐induced artifacts, and reliable signal acquisition at ultrashort echo times.

The calibration model derived from a single experiment generalized well to a second, independent dataset. RMSE values between MR‐derived and sensor‐measured temperatures ranged from 0.93°C to 1.73°C, with an overall RMSE of 1.33°C. The Bland–Altman analysis confirmed good agreement, with a bias near zero (−0.08°C) and narrow limits of agreement. These results are comparable to, or better than, previous studies using magnitude‐based temperature estimation in frozen tissue [13, 15]. However, it should be noted that due to limitations in cryogen delivery within the experimental setup, temperatures below approximately −20°C were not reached, whereas Overduin et al. reported measurements down to −40°C. Accordingly, magnitude noise–bias correction (as used by Overduin et al.) was not performed prior to normalization. Within this temperature/SNR regime, application of a standard Rician debiasing step did not materially change the fitted parameters or RMSE in pilot tests. While this limitation should be addressed in following studies, −20°C still is the clinically relevant threshold where most tissue will become necrotic [9].

A key advantage of the FLORET approach is its ability to achieve true 3D temperature mapping at clinically relevant temporal resolution. The compact frequency spectrum of the trajectory also enhances robustness to eddy currents, which is especially important at short echo times and with rapid gradient switching.

Several limitations of this study should be acknowledged. First, experiments were performed in ex vivo tissue under controlled conditions; in vivo validation is needed to evaluate the effects of perfusion, physiological motion, and tissue heterogeneity. Second, the proposed method is inherently limited to estimating temperatures below 0°C and does not provide information until freezing initiates within a voxel. Finally, the signal–temperature relationship is expected to depend on tissue type, imaging parameters, and magnetic field strength, which may limit the generalizability of the calibration model. The dependence on imaging parameters could potentially be mitigated by acquiring multiple echoes and deriving temperature from changes in $T_{2}^{*}$, although this approach would result in increased scan time [13].

Despite these limitations, the findings suggest that FLORET UTE imaging provides a promising framework for volumetric MR thermometry during cryoablation. Future studies should explore integration into interventional workflows, assess robustness across tissue types, and evaluate performance in vivo under clinical conditions.

Currently, image reconstruction is performed offline (≈30 s per volume on the workstation used), but real‐time processing could be achieved by compressing coil data to six virtual channels and leveraging a NVIDIA RTX A6000 GPU. Temperature‐mapping accuracy was not materially altered by six‐channel compression in pilot tests.

## 5 Conclusion

This study demonstrated that FLORET UTE imaging enables quantitative 3D temperature mapping of frozen tissue during MR‐guided cryoablation with high temporal resolution. The proposed method, based on a voxel‐wise exponential model of signal intensity, produced temperature estimates that showed good agreement with fiber‐optic sensor measurements in ex vivo liver tissue. Compared to previously reported approaches, the FLORET sequence allowed for faster volumetric acquisition without compromising signal quality. While limited to subzero temperatures and evaluated only in an ex vivo setting, the method presents a promising step toward real‐time thermometric monitoring of cryoablation procedures.

## Conflicts of Interest

The authors declare no conflicts of interest.

## Supporting information


**Figure S1:** A representative axial slice over time showing that slight deformation of the phantom occurred. The red arrow points to a vessel where this deformation can be seen especially well.

## Data Availability

The data that support the findings of this study are available from the corresponding author upon reasonable request.
