# Supplementary figures and images for: Temperature Mapping During MR‐Guided Cryoablation Using a FLORET UTE Sequence
Source: Magn Reson Med. 2025 Oct 24;95(3):1653–9. doi: 10.1002/mrm.70152 (PMC12746407; doi:10.1002/mrm.70152)

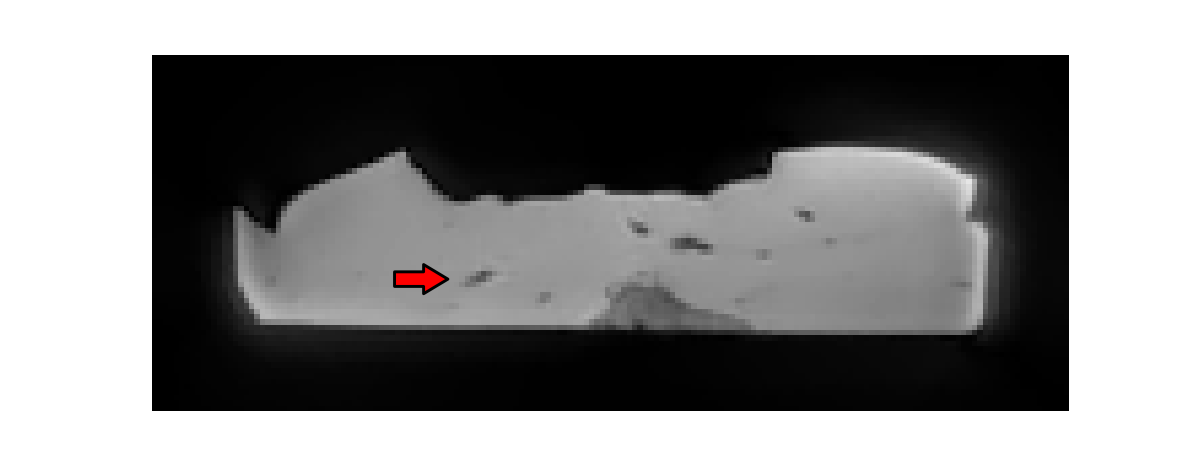

Supplement: Supplementary file 1 — Figure S1: A representative axial slice over time showing that slight deformation of the phantom occurred. The red arrow points to a vessel where this deformation can be seen especially well. [file MRM-95-1653-s001.gif]
